# Time trends and changes in the distribution of malaria cases in the Brazilian Amazon Region, 2004-2013

**DOI:** 10.1590/0074-02760160263

**Published:** 2016-12-01

**Authors:** Isac da SF Lima, Oscar MM Lapouble, Elisabeth C Duarte

**Affiliations:** 1 Universidade de Brasília, Faculdade de Medicina, Programa de Pós-Graduação em Medicina Tropical, Brasília, DF, Brasil; 2 Organização Pan-Americana da Saúde-Brasil, Brasília, DF, Brasil; 3 Universidade de Brasília, Faculdade de Medicina, Brasília, DF, Brasil

**Keywords:** malaria incidence, Brazilian Amazon, joinpoint regression

## Abstract

Recent efforts to reduce malaria incidence have had some successes. Nevertheless, malaria persists as a significant public health problem in the Brazilian Amazon. The objective of this study was to describe changes in malaria case characteristics and to identify trends in malaria incidence in the Brazilian Amazon. This study used data from the Malaria Epidemiological Surveillance and Case Notification Information System from 2004 to 2013. The annual parasite incidence (API) was calculated and joinpoint regression was used to assess the trends in API over time. There was a sharp increase in API in the state of Acre, followed by two periods of decrease. Pará also presented inconsistent decreases over the study period. Amapá, Amazonas, Rondônia, and Roraima showed statistically significant decreases over the period. The sharpest decrease occurred in Rondônia, with a reduction of 21.7% in the average annual percent change (AAPC) (AAPC: -21.7%; 95% confidence interval: -25.4%, -17.8%; p < 0.05). This panorama of malaria incidence highlights the importance of integrating evidence-based malaria surveillance and control. Malaria is highly preventable, and eliminating its transmission should be a goal in coming decades.

Over the last 15 years (2000-2015), there has been an 18.0% reduction in the number of malaria cases and 48.0% reduction in the number of malaria deaths worldwide (WHO 2015a). Despite this success, it is estimated that approximately 3.3 billion people are still at risk of acquiring the disease, 1.2 billion of whom are at high risk of infection (WHO 2014). In 2015, there were 214 million new malaria cases and approximately 438,000 malaria-related deaths in the world (WHO 2015a).

Regarding the geographical distribution of the disease, 88.0% of all malaria cases occur on the African continent, south of the Sahara desert, and approximately 0.3% occur in the Americas (WHO 2015a). In South America, 37.0% of all malaria cases are registered in Brazil, with the vast majority (99.0%) of these concentrated in the Brazilian Amazon Region (BAR). In 2014, a total of 143,442 new malaria cases were reported in this region, representing a reduction of 19.0% in the number of malaria cases compared to 2013 (MS/SVS 2015). This was the lowest incidence reported for the region in the past 35 years (Ferreira & Castro 2016). Fluctuations in malaria transmission, however, have been observed in the past few decades; for example, periods of large-scale epidemics that occurred at the end of the 1990s and in mid-2005 were followed by periods of reduced transmission in the past decade. These reductions may be a result of implementation of the intensification plan of malaria control activities in the Brazilian Amazon (PIACM), which was launched in 2000 (MS/SVS 2000). The extent to which vector control measures have been intensified and the improvements in access to timely diagnosis and treatment (Oliveira-Ferreira et al. 2010, Griffing et al. 2015), as well as the decreasing deforestation rate, may reflect in changes in malaria incidence in the BAR (Guimarães et al. 2016).

In November 2015, Brazil received the Malaria Champions of the Americas Award from the Pan-American Health Organization (PAHO) in recognition of the great achievements in preventing malaria and reducing incidence and mortality associated with the disease. In the same year, the National Malaria Control Programme (NMCP) of the Ministry of Health launched the Plan for Elimination of Malaria in Brazil. This plan is aligned with the United Nations’ Sustainable Development Goals, with a primary objective to reduce the worldwide number of malaria cases by 90.0% and to eliminate the disease in 35 countries by 2030 (WHO 2015b). The Brazilian plan focuses on the elimination of *Plasmodium falciparum* (Ferreira & Castro 2016).

Other studies have described trends in malaria reduction in Brazil (Lapouble et al. 2015) and in the Americas (Carter et al. 2015). The latter study investigated malaria trends over the past five decades in 21 endemic countries, including Brazil. However, no study has examined demographic and other changes in malaria cases or trends in malaria incidence across states in the BAR.

The objective of this study was to describe changes in malaria cases in terms of demographics, socio-economics, and malaria-related variables in selected states of the BAR and to assess trends in malaria incidence (annual parasite incidence - API) between 2004-2013.

## MATERIALS AND METHODS

*Study design* - This epidemiological trend analysis considered both the total number of malaria cases reported in selected states of the BAR and the API (per 1,000 inhabitants) estimated for each state between 2004 and 2013.

*Study population* - The study population included all symptomatic incident malaria cases reported in the states of Acre, Amapá, Amazonas, Pará, Rondônia, and Roraima between 2004-2013. In addition, at an ecological level, the API was estimated for each of the states studied (n = 6) for each of the years analysed (n = 10).

*Selection criteria for Brazilian Amazon states* - The BAR is a large geographical area in Brazil, containing nine of the 27 states in the country. These nine states comprise approximately 60.1% of the Brazilian territory, but only 12.0% of the total population (IBGE 2010). Most malaria cases are concentrated in six states in the BAR (Acre, Amapá, Amazonas, Pará, Rondônia and Roraima). These states account for nearly 98.0% of all incident malaria cases in the Brazilian Amazon (MS 2013, Griffing et al. 2015). The states of Mato Grosso, Tocantins and Maranhão were not included in the study because, together, they account for only 2.0% of all incident malaria cases reported in the country.

The six states share some common characteristics such as low population density compared to states in other regions of Brazil. Roraima has the lowest population density (2.0 inhabitants/km^2^) among the selected states. The percent of the population living in rural areas ranges from 10.2% in the state of Amapá to 31.5% in the state of Pará (IBGE 2010). Although each selected state has distinct economic activities, the economy of the whole region relies mainly on vegetable and mineral extraction (including oil and gas), agriculture, and tourism.

*Epidemiological data* - Incident malaria cases registered in the state of residence between 2004 and 2013 were obtained from the Malaria Epidemiological Surveillance and Case Notification Information System (SIVEP-Malaria). The SIVEP-Malaria system is a data repository under the National Malaria Prevention and Control Programme in the Ministry’s Health Surveillance Secretariat. Under this system, compulsory notification of results (positive or negative), including tests performed on residents and non-residents, of all malaria tests performed by any health service (public or private) in the BAR is required.

*Demographic data* - This study considered the population estimates produced by The Brazilian Institute of Geography and Statistics (IBGE) for each state and year of investigation, except for 2010, when the population size was obtained from the 2010 demographic census.

*Study variables* - The API is an indicator widely used to monitor malaria transmission in Brazil and to estimate the risk of infection (MS/SVS 2002). For the purpose of this study, the API was calculated as follows:


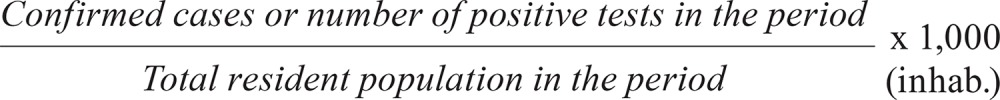


The API was calculated for all cases attributable to *Plasmodium* (*P.*) species as well as those attributable only to *P. falciparum*.

For the purpose of this study, the terms “incident malaria cases” or “malaria incidence” are used synonymously to refer to the number of new malaria cases reported for residents of the six selected states. It is calculated based on the sum of all positive laboratory malaria tests performed on the study population, excluding tests reported as cure verification smear tests (CVS). These are tests performed on people who have had malaria in the last 40 days (for cases of *P. falciparum* infection) or in the last 60 days (for cases of *P. vivax* infection) (MS/SVS 2010), and who are repeating the test to verify whether they have been cured. In this case, it is assumed that these are not new infections; therefore, CVS results were not included in the study analysis.

*Other variables of interest* - Incident malaria cases were described by demographic, socio-economic, and malaria-related variables. These variables were categorised by calculating frequencies and percentages and identifying outliers and missing values. Additionally, when possible, categories were defined based on other studies. Details of the variables analysed follow.

*Demographic variables* - (a) age: 100 years was assumed to be the maximum possible age. Ages were later categorised into groups of 0-5 years, 6-14 years, 15-29 years, 30-59 years, and 60 years or over; (b) sex: women and men; (c) state of residence: Acre, Amapá, Amazonas, Pará, Rondônia, and Roraima; and (d) year of case notification: from 2004 to 2013.

*Socio-economic variables* - (a) level of schooling: no schooling to 5th grade not completed, completed 5th grade to completed 9th grade, high school not completed and higher, and not applicable and no information; (b) type of occupation: agriculture, traveller/tourism, livestock farming/crop production/hunting and fishing/bridge building/mining, domestic service, prospecting, other, and no information or not applicable. The “not applicable” categories in the schooling and occupation variables include all children under six years of age. It was assumed that children in this age group would not have a defined level of schooling or a profession.

*Malaria-related variables* - (a) type of malaria: *P. falciparum* caused by *P. falciparum*, *P. falciparum* + *P. falciparum* gametocytes, and *P. falciparum* gametocytes or *P. falciparum* + *P. malariae*; *P. vivax* caused by *P. vivax* or non-*P. falciparum*, and “mixed” caused by *P. falciparum* + *P. vivax* or *P. vivax* + *P. falciparum* gametocytes; (b) parasite density: +/2, +, ++, +++ or more, and no information. The malaria parasite density was categorised according to the plus system, where the more plus signs (+), the higher the parasite density; and (c) type of detection: passive detection when the patient came to the health facility for malaria testing and active detection when health professionals searched for malaria cases and tested the patients wherever they were.

*Data analysis* - The descriptive analysis of the data involved calculating the frequencies of the demographic, socio-economic, and malaria-related variables, as well as calculating the proportional distribution of the incident malaria cases associated with these variables of interest through the time series, enabling changes over the study period to be visualised. A similar analysis was performed at the state level to investigate how much each state contributed to the total number of malaria cases per year of notification.

The risk of malaria infection was defined as the malaria API estimated for each state and year of notification. This measure enables a comparison between states in relation to their risk of malaria infection (whether it increased or reduced) over the decade studied.

Joinpoint regression was used to identify significant changes in API trend lines between 2004 and 2013. This methodology was developed to analyse time series and uses joinpoint regression to fit the simplest joinpoint model that the data permits. The regression tests whether models with more joinpoints explain trends better than the simplest model (a straight line). Monte Carlo permutation is the method used to test significance, and the variance was estimated by Poisson models (Kim et al. 2000). More details on joinpoint regression are available at https://surveillance.cancer.gov/joinpoint/.

The best model, therefore, is the one that best represents trends through the years, but with the least joinpoints. Because few years were analysed in this study, no more than two joinpoints were allowed for each regression analysis. The annual percent change (APC) and the average annual percent change (AAPC) were estimated from a linear regression of the natural logarithm of the API, the year of notification as the independent variable (explanatory variable), and the state of residence as the stratification variable. The APC identifies those years in which a significant change in the API was observed within the period of investigation, whereas the AAPC identifies the overall trend in the API throughout the entire time series (Clegg et al. 2009). Statistical significance was set to 0.05, and 95% confidence intervals (95% CI) were calculated and reported as needed.

Database manipulation, frequency analysis, and API calculations were performed using SAS/STAT. The joinpoint analysis was conducted using Joinpoint Regression software (version 3.5.1) provided by the Surveillance Research Program, National Cancer Institute, Maryland, United States.

*Ethical considerations* - The National Malaria Prevention and Control Programme formally authorised access to the SIVEP-Malaria database, whilst ensuring confidentiality and non-disclosure of information that could be used to identify those whose data were maintained in the database. This study was approved by the University of Brasília Faculty of Medicine Research Ethics Committee (report number 908.006 dated 25/11/2014) and it fully respected the principles of National Health Council Resolution 196/96.

## RESULTS

A total of 3,365,298 positive malaria test results over the 10-year period were included in the statistical analysis. Overall, a decrease in incident malaria cases could be seen in the BAR over the years of investigation. In general, approximately 64.0% (n = 2,154,391) of all malaria cases were reported in the first half of the study period (2004-2008), compared to 36.0% reported in the second half. Only 4.4% of cases were reported in the last year of investigation.

**Fig. 1:**
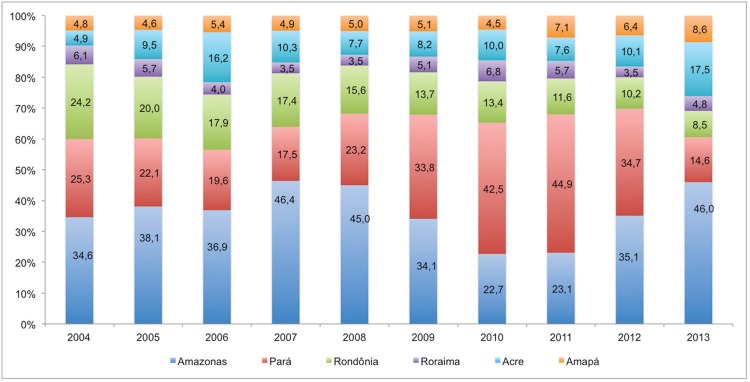
: proportional distribution of malaria cases by state and year of notification, Brazil, 2004-2013.

**Fig. 2:**
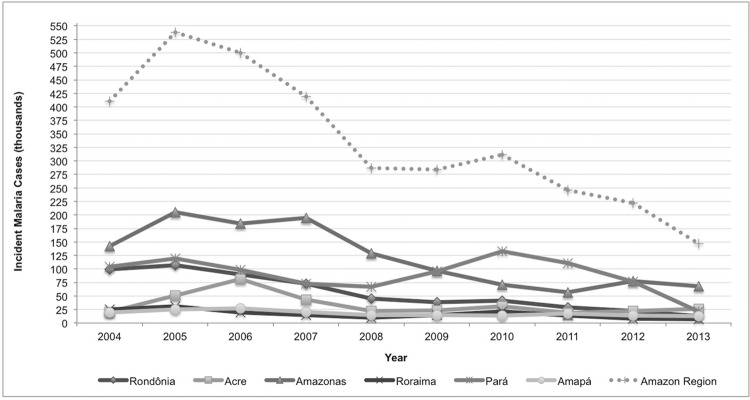
: incident malaria cases in selected Brazilian Amazon states, 2004-2013.

In 2004, nearly 31.6% of malaria cases were children under 15 years of age (Table I), and this proportion remained relatively constant over the study period. In 2013, for instance, this age group accounted for 32.5% of all reported malaria cases. In terms of sex, a gradual increase in the proportion of women among malaria cases was observed over the years. In 2004, women accounted for 35.0% of reported cases of malaria, compared to 2013 when they accounted for 39.2% of all malaria cases. With regard to the proportional distribution of incident malaria cases across the time series and states of residence, there was a reduction in the proportion of malaria cases registered in Rondônia, Pará and Roraima. However, there was a higher proportional distribution of incident malaria cases registered in the states of Acre, Amazonas and Amapá (Table I, Fig. 1). The overall incidence fluctuated through the years, because each state contributed differently to the total number of malaria cases over the study period. The state of Rondônia, for example, accounted for 24.2% of all malaria cases in the year 2004, compared to 8.5% in 2013. In contrast, the state of Acre began the study period with a proportional distribution of malaria cases of 4.9%, but this increased to 17.5% of all malaria cases reported in 2013 (Fig. 1). It is important to mention, however, that all states experienced a substantial reduction in the absolute incidence of malaria over the period of investigation (Fig. 2).

**TABLE I:** Proportional distribution of incident malaria cases based on selected variables and in states of the Brazilian Amazon Region, 2004-2013

Incident malaria cases (N = 3,365,298)	Malaria cases (%^a^)									

	2004	2005	2006	2007	2008	2009	2010	2011	2012	2013

	410,596	537,690	500,255	418,767	287,083	284,271	311,446	246,383	221,869	146,938
Demographic variables (%)										
Age group										
0 - 5 years	12.0	12.9	13.4	13.3	14.1	13.9	13.4	12.9	12.8	11.2
6 - 14 years	19.6	21.5	22.1	22.2	22.4	22.6	22.1	21.8	22.2	21.3
15 - 29 years	34.8	33.4	32.7	32.1	31.4	31.3	31.3	31.4	31.1	31.9
30 - 59 years	30.4	28.9	28.5	29.0	28.8	29.0	29.9	30.5	30.3	31.7
60 years or over	3.1	3.3	3.3	3.3	3.3	3.2	3.3	3.4	3.6	3.9
Sex										
Women	35.0	37.0	37.9	38.3	38.6	38.4	38.5	38.6	38.5	39.2
Men	65.0	63.0	62.1	61.7	61.4	61.6	61.5	61.4	61.5	60.8
State of residence										
Acre	4.9	9.5	16.2	10.3	7.7	8.2	10.0	7.6	10.1	17.5
Amapá	4.8	4.6	5.4	4.9	5.0	5.1	4.5	7.1	6.4	8.6
Amazonas	34.6	38.1	36.9	46.4	45.0	34.1	22.7	23.1	35.1	46.0
Pará	25.3	22.1	19.6	17.5	23.2	33.8	42.5	44.9	34.7	14.6
Rondônia	24.2	20.0	17.9	17.4	15.6	13.7	13.4	11.6	10.2	8.5
Roraima	6.1	5.7	4.0	3.5	3.5	5.1	6.8	5.7	3.5	4.8
Socio-economic variables (%)										
Level of schooling										
No schooling to incomplete 5th grade	42.8	40.6	38.8	39.1	38.5	39.9	38.7	33.7	31.7	29.6
Complete 5th grade to complete 9th grade	27.5	25.3	26.8	30.8	30.8	29.8	33.3	34.3	37.0	38.8
Incomplete high-school to beyond	2.5	2.6	2.9	3.8	3.3	3.0	2.8	7.3	11.4	15.6
Not applicable	15.2	16.4	17.0	16.8	17.9	17.6	16.8	16.2	16.1	14.2
Not informed	12.0	15.1	14.5	9.5	9.6	9.7	8.4	8.5	3.8	1.8
Type of occupation										
Agriculture	24.8	21.9	20.9	22.8	21.8	20.5	18.2	18.8	16.7	16.0
Traveller /tourism	1.5	1.7	1.0	1.0	1.0	1.1	2.0	2.1	2.1	1.8
Livestock farming/crop production/hunting and fishing/bridge building/mining	4.1	3.7	3.5	3.7	4.2	5.2	5.7	6.5	5.0	3.4
Domestic	8.0	7.9	7.2	8.6	8.5	8.3	9.8	10.2	9.2	9.2
Prospector	4.5	3.0	3.0	3.5	4.2	5.0	5.2	4.8	5.7	7.9
Other	21.9	26.9	26.1	27.6	28.4	27.4	24.3	33.1	42.4	45.8
Not informed or not applicable	35.2	34.8	38.3	32.6	32.0	32.4	34.8	24.6	18.9	15.9
Malaria-related variables (%)										
Type of Malaria										
*Plasmodium falciparum*	22.6	23.8	24.7	18.8	14.2	15	13.9	11.7	12.7	15.6
*P. vivax*	76.1	74.8	73.7	80.1	84.9	84	85	87.2	85.7	83
Mixed	1.3	1.3	1.6	1.1	0.9	1	1	1.1	1.5	1.4
Parasite density (grade as number of (+) signs)										
+/2	39.5	42.1	42.6	40.4	39.0	37.0	37.2	36.1	37.1	42.1
+	23.3	22.5	21.9	22.4	22.0	21.1	20.3	19.0	18.9	18.9
++	34.4	32.5	33.0	34.5	36.0	39.0	39.6	41.4	38.7	34.4
+++ or more	2.7	2.9	2.5	2.6	3.0	2.9	2.9	3.3	3.3	2.6
Not informed	-	-	-	-	-	-	-	0.2	1.9	2.0
Type detection										
Passive detection	79.3	76.3	73.9	75.7	76.2	75.6	76.3	79.5	78.6	75.4
Active detection	20.7	23.7	26.1	24.3	23.8	24.4	23.7	20.5	21.4	24.6

*a*: column percentages within each variable category.

Regarding socio-economic characteristics, a change in the proportional distribution of incident malaria cases was observed, based on the level of schooling. An increase in the proportion of individuals with higher levels of education among malaria cases was observed through the years of the study. For instance, illiterate people or those with schooling up to the 5th grade (4th year) accounted for 42.8% of reported malaria cases in 2004, but in 2013 the low schooling population accounted for only 29.6% (a 30.0% reduction) of the reported malaria cases. In contrast, people with high school not completed and higher increased among malaria cases, from 2.5% in 2004 to 15.6% in 2013 (Table I).

Regarding occupation, the contribution of agricultural workers to total malaria cases decreased between 2004 and 2013 (24.8% and 16.0%, respectively), whereas the contribution of prospectors increased from 4.5% in 2004 to 7.9% in 2013. There was a noteworthy increase in the proportion of cases in the occupation category “other” (from 21.9% to 45.8%) and a marked decrease in the proportion of cases categorised as “no information or not applicable” (from 35.2% to 15.9%) between 2004 and 2013, respectively, probably indicating changes in the notification process. This will be addressed in the discussion section.

In terms of malaria-related characteristics, the proportion of incident malaria cases infected by *P. falciparum* decreased from 22.6% in 2004 to 15.6% in 2013, whereas the proportion of cases with *P. vivax* infections increased from 76.1% to 83.0%. Additionally, the proportion of individuals in whom malaria was identified through active detection (household visit) showed a small but important increase between the years 2004-2013 (20.7% and 24.6%, respectively).

**Fig. 3:**
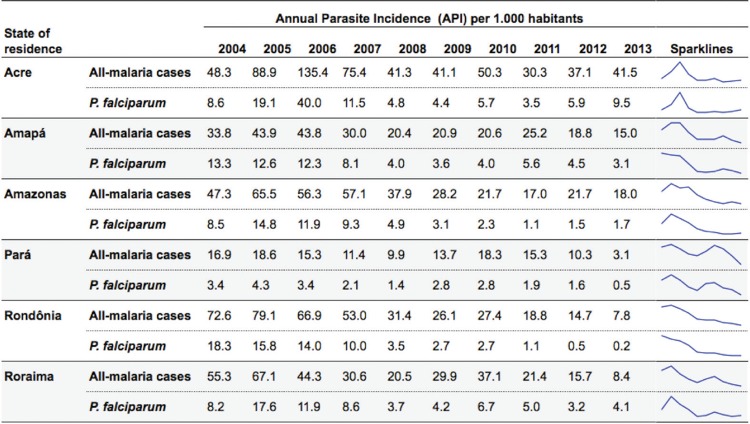
: time series of all malaria cases and *Plasmodium falciparum* annual parasite incidence by state and year of notification, Brazil, 2004-2013.

Fig. 3 shows the API for each of the six states across the time series, and its respective Sparkline Graphic, whereas Table II shows the results of the joinpoint regression. The API among all malaria cases increased in the state of Acre (APC: 127.3%; 95% CI: -10.7%, 478.9%) between the years of 2004-2006. The API increased from 48.3 cases per 1,000 inhabitants to 135.4 cases. From 2006 on, the API decreased on two different occasions. The first downward trend occurred between 2006-2008 (APC: -40.3%; 95% CI: -76.6%, 51.9%), whilst the second took place between 2008-2013 (APC: -2.9%; 95% CI: -21.2%, 19.7%). Nevertheless, neither of these two decreases were statistically significant, possibly due to the small sample (n = 10, study period) and consequent lack of power. This, also, will be considered in the discussion section. Among *P. falciparum* cases, there was an increase in the API between 2009-2013 (APC: 21.3%; 95% CI: -12.2%, 67.5%), whereas the all-malaria API decreased by -2.9% between 2008-2013.

**TABLE II:** Trends determined by a joinpoint regression of the malaria annual parasite incidence by state of residence, Brazil, 2004-2013

State of residence	All-malaria cases							

	APC				AAPC			
	
	Time period	APC	95% CI		Trends	AAPC	95% CI	
Acre	2004-2006	127.3	-10.7	478.9	2004-2013	-2.3	-20.0	19.3
	2006-2008	-40.3	-76.6	51.9				
	2008-2013	-2.9	-21.2	19.7				
Amapá	2004-2013	-9.8*	-14.3	-5.2	2004-2013	-9.8*	-14.3	-5.2
Amazonas	2004-2013	-14.4*	-19.1	-9.4	2004-2013	-14.4*	-19.1	-9.4
Pará	2004-2008	-12.5	-30.2	9.8	2004-2013	-18.2*	-28.1	-6.9
	2008-2012	17.1	-18.2	67.7				
	2012-2013	-70.4*	-85.6	-39.4				
Rondônia	2004-2013	-21.7*	-25.4	-17.8	2004-2013	-21.7*	-25.4	-17.8
Roraima	2004-2013	-16.5*	-23.0	-9.6	2004-2013	-16.5*	-23.0	-9.6

	*Plasmodium falciparum*							

Acre	2004-2006	102.0	-52.3	755.7	2004-2013	-1.4	-29.2	37.4
	2006-2009	-61.7	-91.0	62.3				
	2009-2013	21.3	-12.2	67.5				
Amapá	2004-2013	-14.8*	-21.2	-7.9	2004-2013	-14.8*	-21.2	-7.9
Amazonas	2004-2006	57.8	-2.7	155.9	2004-2013	-16.6*	-24.0	-8.6
	2006-2011	-38.1*	-44.4	-31.0				
	2011-2013	17.3	-27.7	90.3				
Pará	2004-2013	-14.0*	-22.4	-4.6	2004-2013	-14.0*	-22.4	-4.6
Rondônia	2004-2013	-38.4*	-43.6	-32.8	2004-2013	-38.4*	-43.6	-32.8
Roraima	2004-2013	-13.1*	-21.0	-4.3	2004-2013	-13.1*	-21.0	-4.3

APC: annual percentage change; AAPC: average annual percentage change; CI: confidence interval; *: statistical significance: p < 0.05.

Statistically significant decreases in the API were found for the states of Amapá, Amazonas, Rondônia and Roraima between 2004-2013. In these four states, the model that best represented the decrease in API did not include any joinpoints, which means that the reductions could be represented by a straight line with a negative slope for the entire period assessed. The model estimated the following downward AAPC (AAPC for these states: Rondônia -21.7% (95% CI: -25.4%, -17.8%; p < 0.05); Roraima -16.5% (95% CI: -23.0%, -9.6%; p < 0.05); Amazonas -14.4% (95% CI: -19.1%, -9.4%; p < 0.05); and Amapá -9.8% (95% CI: -14.3%, -5.2%; p < 0.05). In the states of Amapá, Amazonas and Rondônia, reductions in API among *P. falciparum* cases were more relevant compared to the API among malaria cases, during the study period.

The states of Pará and Acre showed variations in the API trend based on all malaria cases, with two joinpoints in the study period. In the state of Pará, the API showed a decreasing trend (APC: -125.0%; 95% CI: -30.2%, 9.8%) between 2004-2008, followed by an increasing trend between 2008-2012 (APC: 17.1%; 95% CI: -18.2%, 67.2%). Despite the magnitude of these changes in the API, neither change was statistically significant. After 2012, however, the all-malaria API decreased from 10.3 cases per 1,000 inhabitants to 3.1 cases, representing a statistically significant change of -70.4% in APC (APC: -70.4%; 95% CI: -85.6%, -39.4%; p < 0.05). The *P. falciparum* API reduction was linear for the state of Pará, with an AAPC of -14.0% (AAPC: -14.0%; 95% CI: -22.4%, -4.6%; p < 0.05). On the other hand, the all malaria API in the state of Amazonas showed an APC reduction of -14.4% between 2004-2013, whereas the *P. falciparum* API increased between 2004-2006, dropped 38.0% between 2006-2011, and then increased again by 17.3% between 2011-2013.

It is noteworthy that, with the exception of Acre, all of the other states showed statistically significant reductions in API (all-malaria and *P. falciparum*-specific) over the ten years of the time series.

## DISCUSSION

This study described diverse API trends in selected states of the BAR: Rondônia showed a sustained reduction in this indicator (-21.7%); Amapá, Amazonas, and Roraima also showed sustained reductions, although with relatively low magnitudes ranging from -9.8% to -16.5%; Pará showed a reduction of -70.4% only in the last two years of the time series (2012-2013); whereas Acre showed no significant reduction in the indicator. This study also demonstrated a change in the profile of malaria cases reported over the study period. Among the malaria cases, there was an increase in the proportion of women; people living in the states of Acre, Amapá, and Amazonas; people with more years of formal education; people working as prospectors; malaria caused by *P. vivax*; and cases identified through active detection. The variables age and parasite density did not change with regard to their contributions to malaria cases over the study years.

The increasing proportion of people with higher levels of education among the reported malaria cases may reflect, to a large extent, the average increase in schooling that has occurred in the Brazilian population in recent decades. Literacy among youth and adults (people aged 15 and over) increased from 86.7% (1999) to 91.3% (2012), and the rate of functional illiteracy in the same age group declined from 27.3% (2001) to 18.3% (2012), representing a 33.0% decrease (MEC 2014). Moreover, in some regions, migration from rural to urban and suburban areas that were receptive to the vector and the disease may have contributed to establishing malaria transmission in these locations and changed the population affected. This may account for the differences in malaria case characteristics through time documented in this study, especially the greater contribution of women and people with more years of education and the proportional increase in intra-household transmission of malaria, which is more frequent in urban and suburban areas (Costa et al. 2010).

Regarding occupation, the proportional increase in cases self-reporting their occupation as “other” is noteworthy. Some occupational categories may not yet have been included in the SIVEP-Malaria system in more recent years, resulting in the misclassification of certain occupations into the “other” category. On the other hand, there was a gradual but consistent reduction in cases self-reporting their occupational type as “agriculture”. Future studies should examine this result in more detail.

This study also found an increase in the proportion of incident malaria cases diagnosed through active detection. Many factors might have contributed to this change, such as the hiring and training of more professionals for malaria control and the purchase and use of rapid tests to diagnose malaria (Ferreira & Castro 2016), as well as the decision to perform active tracing to detect symptomatic and oligosymptomatic cases (Costa et al. 2010).

*Trends in Acre* - Between 2004 and 2006, the state of Acre showed an increase in API with an APC of 127.3%. After this, the state reported two periods of API reduction, the first and sharper reduction was observed between 2006-2008 (APC: -40.3%) and the second was observed between 2008-2013 (APC: -2.9%). Neither of these trends, however, was statistically significant, despite their magnitudes. Epidemics occurring in Acre, particularly in the municipality of Cruzeiro do Sul between 2004-2006, have already been documented in the literature (Costa et al. 2010, Braz et al. 2012). The results of another study conducted in Acre suggest that fish farming contributes to the high levels of malaria transmission in the region, because an abundance of anopheline mosquitoes were observed within 100 metres of fishponds and the rate of malaria notifications in this location increased over time (Reis et al. 2015a). Despite the fact that both natural water bodies and fishponds can be infested with immature (aquatic-phase) mosquitoes, it is important to note that the water in fishponds is on average four times more infested with anopheline larvae than that from natural water bodies (Rodrigues et al. 2008, Reis et al. 2015b).

Fish farming was heavily subsidised by the government of the state of Acre in 2005, a possible reason for the explosion in malaria cases in this state, particularly in 2006 (Costa et al. 2010, Oliveira-Ferreira et al. 2010, Duarte et al. 2014) and in 2012, when Acre was one of the three states with the highest rates of deforestation and malaria (Guimarães et al. 2016). The complex scenario of malaria surveillance on the border between Brazil, Bolivia and Peru deserve special attention. Differences in malaria surveillance along the border, lack of trained personnel and malaria specialists, and lack of knowledge about malaria and its prevention among the local population are just a few issues that have been reported in the literature (Peiter et al. 2013) and that contribute to on-going malaria transmission and incidence in the state.

The first and sharpest reduction in malaria incidence in the state of Acre is thought to have occurred in response to large-scale efforts to recruit and train local health teams, as well as to increase timely diagnosis and the distribution of free treatment within 48 h of symptom onset (Griffing et al. 2015). Insecticide-treated mosquito nets were also distributed in the municipality of Cruzeiro do Sul beginning in December 2007 as one of the main anti-malarial strategies in the state (Costa et al. 2010). These initiatives led the state to receive successive prizes from the Pan American Health Organization (PAHO 2013). The reduction in API, based on all malaria cases or *P. falciparum* cases, in the state of Acre was not statistically significant, although it may be epidemiologically relevant.

*Trends in Amapá* - The state of Amapá showed a sustained and statistically significant reduction in API between 2004-2013. The average annual percent reduction for all malaria cases and for *P. falciparum* cases only was relatively low throughout the period (AAPC: -9.8%, p < 0.05 and AAPC = -14.8%, p < 0.05,). Amapá has a relatively small population (770,000 inhabitants) and much of it (57.0%) is concentrated in the capital (IBGE 2010). Improving access to health care services and distribution of insecticide-treated mosquito nets were some of the strategies implemented by the state and municipal governments for malaria control and incidence reduction (CVS 2015). Additionally, there is a low-density population in non-urban areas (72.0% of the state’s territory), and consequently malaria cases might be highly dispersed. The reduction in malaria incidence might have been even more significant if not for an international border region where malaria is endemic, particularly in the border area between the municipality of Oiapoque (Amapá) and Saint-Georges town in French Guyana, where illegal prospecting, unstable healthcare, and a constant flow of people represented important challenges for malaria control in this state. The fact that Amapá has a endemic area for malaria (the international boarder), unstable healthcare and constant flow of people are all challenges for malaria control in the state. These events potentially prevented the state to have had even higher reduction in API that was observed in the study results. In other words, over and beyond these problems, the state had a decrease in API (a modest decrease) over the study period (Braz et al. 2013).

*Trends in Amazonas* - Similar to the state of Amapá, Amazonas also showed a statistically significant and constant reduction in API. A small but sustained all-malaria API reduction (AAPC = -14.4%; p < 0.05) was also observed in this state between 2004-2013. The average annual percentage reduction for *P. falciparum* cases was 16.6% (p < 0.05) during the study period. A sharp and statistically significant reduction in API was identified between 2006-2011 (AAPC = -38.1%; p < 0.05). Implementation of the PIACM after 2000 probably had some impact on the malaria API in the Brazilian Amazon. The state of Amazonas observed a 71.0% percent decline in malaria incidence between 1999 and 2001, which was the highest among the states in this region (MS/SVS 2003).

As the result of many efforts to reduce malaria incidence and control the vector (Braz et al. 2014), the state of Amazonas showed a 21.7% reduction in the number of epidemic municipalities between 2003-2010. Even with this reduction, the disease incidence remained high. Another study investigating the relationship between malaria incidence and urban expansion in Manaus, the capital of the state, found that areas with high rates of deforestation and urban expansion contributed most to the epidemic profile of malaria, which was re-introduced after 13 years without autochthonous malaria transmission (Saraiva et al. 2009). Manaus accounts for nearly 51% of the state’s population (IBGE 2010). Other factors that may have slowed the reduction in API in the state and contributed to on-going malaria transmission include climatic factors (flooding), geographic factors (limited access to health care in remote areas), deforestation, unplanned human settlements (unauthorised land occupation), and fish farming activities, along with unstable epidemiological and entomological surveillance (Saraiva et al. 2009).

*Trends in Pará* - The time series analysis of data from the state of Pará showed an interesting fluctuation in the API based on all malaria cases between 2004-2013. There was an increase in API between 2008 and 2012 that slowed the average annual percent change reduction that began in 2004. Indeed, between 2010-2011, Pará accounted for most malaria cases reported on the SIVEP-Malaria system (Parente et al. 2012). In addition to the geographic, ecological, and climatic factors that are ideal for mosquito proliferation, the population in Pará also lacks adequate access to health care and health education (Sousa et al. 2015).

Despite these challenges, a statistically significant API reduction was observed between 2012-2013 (APC: -70.4%; 95% CI: -85.6%, -39.4%, p < 0.05). This result is similar to the results of another study that reported an increase in the number of municipalities classified as “low malaria risk” (API < 10) that were previously classified as “middle malaria risk” (10 ≤ API < 50), probably as a result of the increasing availability of health services in remote municipalities (Sousa et al. 2015). Approximately 7.6 million people live in the state of Pará, 18.0% of who live in the capital, Belém (IBGE 2010). In 2014, the Public Health Department of the state issued a press release indicating the intensification of malaria control activities, including the distribution of insecticide-treated mosquito nets to the residents of endemic municipalities. One such municipality was Anajás, which registered only 170 cases of the disease in 2014, compared to more than 3,000 cases in 2011 (Agência Pará 2014). The release also reported improvements in health care and education on the correct use of insecticide-treated mosquito nets. These factors may have contributed to the reduction in API in recent years; however, there are still areas of high disease transmission in the state of Pará that are mainly associated with agriculture (crops and cattle) and mineral exploration activities (Santos et al. 2013).

*Trends in Rondônia* - In the state of Rondônia, a decrease in the AAPC of 21.7% (AAPC: -21.7%; 95% CI: -25.4%, -17.8%; p < 0.05) was observed in the period 2004-2013. In 2004, this state accounted for approximately 24.0% of all malaria cases in the BAR. However, this percentage gradually decreased over the last years of the study period, reaching 8.6% in 2013. Despite the overall decrease in the state’s API, the distribution of malaria cases was highly heterogeneous and included high incidence areas such as the municipalities of Porto Velho and Ariquemes, both traversed by important rivers, in the northern areas (Vieira et al. 2014). One of the factors that may explain the reduction in the state’s API is the malaria action and control plans implemented by the state and municipal governments (AMS 2012) in conjunction with companies (CEMIG 2005) responsible for the construction of two hydroelectric power stations (Santo Antônio and Jirau). These constructions began in mid-2004 in the municipality of Porto Velho. The action plan included vector control, improvements in sanitation and urban conditions, as well as health promotions regarding the need to seek early diagnosis and effective treatment for malaria (Katsuragawa et al. 2009). However, further studies must be conducted to better understand the possible positive impacts on malaria incidence of building these hydroelectric power stations.

An epidemiological study on malaria reported that 58.0% fewer cases and 36.2% fewer relapse/recrudescence cases occurred in 2012 compared to 2008 (Vieira et al. 2014). Socio-economic improvements in the population of Rondônia, with a reduction in low incomes and increase in formal employment, that were observed particularly over the past decade, might have influenced the number of malaria cases (DATASUS 2016). Reduced levels of deforestation, plant exploration, mining, and gold prospecting also may have contributed to this reduction in malaria risk, because these human impacts on the environment are typically associated with higher mosquito concentrations (Teles et al. 2013, Reis et al. 2015b).

*Trends in Roraima* - Roraima showed a clear reduction in API between 2005 and 2008, followed by an increase in this indicator in 2009-2010, when this state accounted for 6.8% of all reported malaria cases in the BAR. Another study conducted in the municipality of Cantá, which tested an artificial neural network to predict the incidence of malaria in this municipality, confirmed the results of this analysis (da Cunha et al. 2010). Between 2011 and 2013, malaria cases began to fall again. Over the entire study period, this state showed a statistically significant decrease (-16.5%; 95% CI: -23.0%, -9.6%; p < 0.05) in the risk of malaria transmission. Roraima has a population of just over 450,000 inhabitants (IBGE 2010) with approximately 63.0% of the total population concentrated in the state capital, Boa Vista. In 2005, several immature specimens of malaria vectors were described in areas surrounding Boa Vista, especially in the dry season, but anopheles darling larvae were found rarely. The authors discuss the usual persistence of malaria transmission even under these conditions (Nagm et al. 2007). Another study showed the impact of human settlements and other anthropogenic changes on the environment and how climate variability affected both population density and relative abundance of these vectors (Gomes et al. 2008). Despite the reduction in API observed in the state of Roraima, it will be very important to study the breeding sites and mosquito population density and diversity in order to improve vector control strategies and maintain control of malaria in endemic areas.

According to a report produced by the State Malaria Control Centre under the Roraima State Health Department, new state policies were implemented in 2005 to supervise, monitor, and carry out malaria control actions jointly with municipal health teams (DSR 2014). These actions, together with a reduction in deforestation and overall socio-economic improvements in the population of Roraima, may have contributed to the reduction in malaria incidence.

Some limitations of this study relate to the use of secondary data (the potential for inaccurate information and omission of relevant variables), the possibility of more than one positive test being performed and reported for the same malaria episode (over reporting), and the possibility that some malaria cases were not reported because of incorrect case diagnosis and/or recording (underreporting). Nevertheless, SIVEP-Malaria is known to be a robust information system. Notification of malaria is compulsory in Brazil, including reporting on both positive and negative tests, performed on residents and non-residents, in both public and private health services. Studies have shown the high capacity (sensitivity) of SIVEP-Malaria in early detection of malaria epidemics in municipalities of the Amazon Region (Braz et al. 2012).

A statistically significant reduction in the API between 2004 and 2013 was observed in five (Amapá, Amazônia, Rondônia, Roraima and Pará) of the six selected states in the BAR. Acre was the only state that did not present a statistically significant reduction, despite a decrease in API after 2006. Some of these states, however, showed variability in malaria incidence, with increases in API during the study period. This geographic and temporal diversity suggests the need to establish integrated and evidence-based malaria surveillance and control strategies in the region, to address determinants of malaria transmission locally and in a sustainable manner.

Although Brazil seems to be progressing toward malaria elimination, there are still many challenges such as the high prevalence of asymptomatic infections, anti-malarial drug resistance in *P. falciparum* and *P. vivax*, the burden of malaria in pregnancy, the need for better vector control strategies, the need for more effective surveillance, and the effects of environmental changes and climatic variability on transmission.

To achieve the Brazilian Plan for Elimination of Malaria, improvements in community sensitisation and education, as well as guidelines on best diagnostic practices, treatments, and vector control strategies, are required. Furthermore, changes in the profile of patients with malaria (e.g., a proportional increase in women and people with more schooling among malaria cases) should inform malaria control actions in the area, raising hypotheses and suggesting possible changes in groups most at risk for malaria infection.

Further efforts need to be made to interrupt malaria transmission in the coming years, principally because of its high potential for prevention. Present and future strategies must be aligned with the Plan for Elimination of Malaria and customised at the regional and municipal levels to account for geographic differences.
